# Distribution characteristics of aerosol microorganisms in bronchoscopy room and the risk assessment of nosocomial infection

**DOI:** 10.3389/fpubh.2025.1556364

**Published:** 2025-04-28

**Authors:** Wen Zhu, Yuehua Xu, Ting Chen, Minhua Shi

**Affiliations:** Department of Pulmonary and Critical Care Medicine, The Second Affiliated Hospital of Soochow University, Suzhou, China

**Keywords:** bronchoscopy, aerosol, microorganisms, respiratory pathogens, nosocomial infection

## Abstract

**Objective:**

A large number of aerosols containing pathogenic microorganisms can be produced during bronchoscopy. The aim of the study is to evaluate the risk of nosocomial infection by pathogenic microorganisms after bronchoscopy. The distribution characteristics of aerosol microorganisms were detected before and after bronchoscopy, and then compared with the distribution characteristics of the patients’ respiratory pathogens.

**Methods:**

A total of 152 patients underwent bronchoscopy in the bronchoscopy room from May 06, 2024 to June 30, 2024. Airborne microorganisms were collected in the bronchoscopy room before and after the bronchoscopy, then cultured, counted and identified, to analyze the species, numbers and changes of microorganisms. At the same time, the data of respiratory pathogens and nosocomial infection of all patients were collected to evaluate the correlation between air microorganisms and respiratory pathogens, and the risk of nosocomial infection.

**Results:**

(1) The concentration of air microorganisms after bronchoscopy was 89.60 ± 63.52 CFU/m^3^, significantly higher than 43.80 ± 26.70 CFU/m^3^ before bronchoscopy (*p* < 0.001). The increase in air microorganism concentration was in accordance with the total number of patients and the number of patients with respiratory infectious diseases on the same day (*p* < 0.001). After bronchoscopy for patients with infectious diseases, the concentration was significantly higher than that for patients with non-infectious diseases (*p* < 0.05). (2) The bacteria accounted for 75.34, 70.35% of the total aerosol microorganisms, fungi 22.17, 26.80% before and after bronchoscopy. The bacteria mainly included *Micrococcus luteus*, *Staphylococcus epidermidis*, *Staphylococcus hominis*, *Haemophilus influenzae*, *Neisseria faunalis*, *Staphylococcus capitis*, etc. The fungi mainly included *Aspergillus flavus*, *Aspergillus niger*, *Saccharomyces albicans*, *Penicillium* spp., etc. (3) The increase in air microorganisms after bronchoscopy was consistent with the distribution of pathogens causing respiratory infections in patients (*p* < 0.001). The increased pathogens were mainly composed of common respiratory pathogens, but it did not increase the risk of nosocomial respiratory infections in patients (*p* = 0.735).

**Conclusion:**

Bronchoscopy can increase the concentration of aerosol microorganisms. The increased microorganisms are related to the respiratory pathogens of patients, which are mainly the common pathogens of pulmonary infection. This, however, does not increase the risk of nosocomial respiratory infection.

## 1 Introduction

Nosocomial infections/Hospital-acquired infections (HAIs) refer to infections that patients acquire within a hospital setting, including those that occur during their hospital stays and those acquired in the hospital but occur after discharge. However, HAIs do not encompass infections that were already present or had begun before admission. Infections acquired by hospital staff in the hospital also belong to HAIs (1). Nosocomial infection is a serious threat to the life and health of patients.

In recent years, aerosol and indoor air transmission issues have become one of the research hotspots, especially after the outbreak of COVID-19. The hospital environment is an important part of the occurrence of nosocomial infection (2–4), while pathogenic microorganisms may easily spread into the air via sneezing, coughing, talking and other ways, polluting the indoor environment of the hospital (5, 6).

Respiratory infection (pneumonia & lower respiratory tract infection) is one of the main components of nosocomial infection (7–9). The increased risk of infection is associated with the severity of the patient’s illness, the length of exposure to invasive devices and procedures, increased patient contact with medical workers, and length of hospital stay.

Bronchoscopy is a common and widely used procedure in the clinical diagnosis and treatment of lung diseases. It enables sampling or therapeutic interventions for lesions in lung lobes and bronchi. As an invasive procedure, it can stimulate the airway mucosa, potentially causing patients to cough or expectorate, thereby generating aerosols which may carry pathogenic microorganisms. This eventually results in significant contamination of the indoor environment (10–13).

In the past, the monitoring of air microorganisms in the bronchoscopy room was mostly in compliance with the total number of colonies under static conditions (14), lacking research on the real-time changes in the number of colonies and the distribution of bacteria and fungi produced by air microorganisms during bronchoscopy operation, as well as the analysis of the correlation with nosocomial respiratory infections.

Therefore, the purpose of this study was to clarify the changes in the distribution characteristics of airborne microorganisms before and after bronchoscopy operation, to compare them with the respiratory tract pathogens of patients, and to evaluate the correlation between aerosol microorganisms and nosocomial respiratory tract infection. Also, the study was designed with the hope of providing evidence for the control of nosocomial infection and improving the prevention and treatment level of nosocomial infection.

## 2 Materials and methods

### 2.1 Main experimental materials

Airborne bacteria sampler MAS-100NT (Merck MBV, Germany), Matrix-assisted laser desorption/ionization time-of-flight (MALDI-TOF) mass spectrometer (Bruker, Germany), thermostatic incubator (Thermol Fisher, USA), agar plates 90 mm (including blood agar plates, chocolate agar plates, MacConkey agar plates, anaerobic blood agar plates).

### 2.2 Sampling and identification of airborne microorganisms in the bronchoscopy room

The experiment was conducted in the bronchoscopy room of the Second Affiliated Hospital of Soochow University. The bronchoscopy room covered an area of about 40 square meters. The plasma air sterilizer (daily 6:00–7:00 and 12:00–13:00, each for 1 h), ultraviolet light (30 min after all bronchoscopy on that day) and surface wiping were routinely used as comprehensive disinfection measures. The indoor temperature was 15°C–25°C, and the relative humidity was 30–60%. The number of bronchoscopy and treatment (including electronic bronchoscopy, ultrasonic bronchoscopy, interventional treatment, etc.) was ≤5 every morning. The disinfection procedures for bronchoscopes were evaluated quarterly in accordance with the guidelines for nosocomial infection management and control. The experiment was conducted from 7:00–12:00, and the samples of air were divided into 2 groups according to the sampling time, which were the pre-bronchoscopy group (7:00–8:00) and post-bronchoscopy group (8:00–12:00, after the bronchoscopy of all patients was completed on the morning of the same day) respectively. Microorganisms in the air were collected in 40 workdays, and the temperature, relative humidity and number of people in the bronchoscopy room (including medical workers, patients and experimenters) were recorded. The number of people in the room was limited to 3–5.

#### 2.2.1 Setting the sampling sites

According to the GB15982-2012 “Hygienic Standards for Hospital Disinfection” of the Ministry of Health of the People’s Republic of China (14), five sampling sites were set in the room with an area of >30 square meters for air quality testing, including four in the corners of the room and one in the center. The horizontal distance between the four corners and the wall was 1 m, and the vertical distance from the ground was 1 m. Each sampling time was no more than 30 min.

#### 2.2.2 Sampling method

The room was cleaned and disinfected each morning before and after the bronchoscopy operation. Before the operation of the bronchoscopy on the same day, which was from 7:00 to 8:00, the microorganisms in the air were collected at 5 sampling sites and recorded as the pre-bronchoscopy group. Then the medical staff began to perform aerosol generation operations such as electronic bronchoscopy and ultrasonic bronchoscopy on the patients, and the microorganisms in the air were collected at the same sampling sites immediately after the bronchoscopy of the last patient, recorded as the post-bronchoscopy group. The experiment was repeated 40 times in total.

#### 2.2.3 Microbial sampling, culture and counting

According to the air sampler method, the airborne bacteria sampler MAS-100NT was used, which was calibrated before use, and the sampling flow rate was 100 L. The sterile agar plate with a diameter of 90 mm was placed in the sampler, and the sampler was placed at each sampling site. By this means, the air bacteria and fungi were directly collected on the agar plates. Blood agar plates, chocolate agar plates, MacConkey agar plates, and anaerobic blood agar plates were used as a combination to culture different aerosol microorganisms. After sampling, the agar plates were placed in the incubator at (36 ± 1)°C for 48 h, and the number of colonies was counted.

The formula “*p* = 1,000 N/V” was used to calculate the total number of colonies in the air (CFU/m^3^), in which p stands for the microbial concentration in the air (CFU/m^3^), N represents the number of colonies in the medium (CFU), and V means the volume of the sampled air (m^3^). The sampling flow of the airborne bacteria sampler used in this experiment was set to 100 L, so *p* = 10 N. Since there were five sampling sites in this experiment, N is the average of the numbers of colonies at the five sampling sites in each group.

The agar plate produced in the same batch was used as a negative control and cultured in the same incubator with the experimental sample and the results were recorded.

#### 2.2.4 Identification of airborne microorganisms

Agar plates were cultured in the incubator at (36 ± 1)°C for 48 h. After colony observation and counting, MALDI-TOF mass spectrometer was used to detect and record the strains in the Examination Center of the Second Affiliated Hospital of Soochow University.

### 2.3 Patient data and sample analysis

#### 2.3.1 Collection of patient data

Inclusion criteria: Patients with complete clinical data who underwent bronchoscopy in the bronchoscopy room of the Second Affiliated Hospital of Soochow University from May 6, 2024 to June 30, 2024, volunteered to participate in this study. Exclusion criteria: Patients who did not undergo bronchoscopy from May 06, 2024 to June 30, 2024, and whose clinical data were incomplete.

A total of 152 patients were enrolled in this study and their clinical data were collected. This study was approved by the ethics committee of the Second Affiliated Hospital of Soochow University, and written informed consent was obtained from each subject.

#### 2.3.2 Collection and identification of respiratory pathogens

Routine sputum and bronchoalveolar lavage fluid (BALF) were collected from patients with bronchoscopy for this study. The collected sputum and BALF samples of everyone were sent to the Examination Center of the Second Affiliated Hospital of Soochow University for bacterial and fungal culture [blood agar plates, chocolate agar plates, MacConkey agar plates, anaerobic blood agar plates, Sabouraud dextrose agar (SDA), and potato dextrose agar (PDA)]. And MALDI-TOF mass spectrometer was utilized for the detection of bacterial and fungal species. At the same time, a part of the BALF samples of certain patients were subjected to metagenomic next-generation sequencing (mNGS) and X-pert *Mycobacterium tuberculosis*/rifampicin (MTB/RIF) to identify and analyze pathogens in accordance with the medical requirements.

### 2.4 Statistical analysis

SPSS 25.0 software was used for data statistics and analysis. Measurement data were expressed as mean ± standard deviation (x ± s) and *T*-test was used. Counting data were expressed as rates and *Chi-square* test or *Fisher* exact probability test was used. *Pearson* correlation analysis was used when the data conformed to the normal distribution. A value of *p* < 0.05 was considered statistically significant.

## 3 Results

### 3.1 Clinical characteristics of the patients

A total of 152 patients underwent bronchoscopy during the period and their characteristics were summarized (Table 1). The mean age was 61.7 ± 15.6 years, and 61.84% of them were male. 86.18% of the patients were inpatients in the general respiratory ward, 13.82% were outpatients in the respiratory department, and 5.92% were inpatients in other wards.

**Table 1:** Clinical characteristics and ratio of the patients (*n* = 152).

Clinical characteristics	Number	Percentage (%)
Age	61.7 ± 15.6	–
Gender		
Male	94	61.84
Female	58	38.16
Inpatient	131	86.18
Respiratory ward	122	80.26
Other wards	9	5.92
Outpatient	21	13.82
Respiratory diseases	152	100
Infectious diseases	100	65.79
Bronchiolitis	4	2.63
Pneumonia	70	46.05
Obstructive pneumonia	6	3.95
Bronchiectasis with infection	5	3.29
Pulmonary tuberculosis	5	3.29
Nontuberculous mycobacteria	2	1.31
Pulmonary aspergillosis	7	4.61
Lung abscess	1	0.66
Non-infectious diseases	52	34.21
Lung cancer	41	26.97
Pulmonary nodules	3	1.97
Sarcoidosis	4	2.63
Bronchial foreign bodies	1	0.66
Bronchial stenosis	2	1.32
Hemoptysis of unknown cause	1	0.66
Antibiotic therapy	107	70.39

Patients were divided into two categories: respiratory infectious diseases (65.79%) and respiratory non-infectious diseases (34.21%). Respiratory infectious diseases encompassed bronchiolitis (2.63%), pneumonia (46.05%), obstructive pneumonia (3.95%), bronchiectasis with infection (3.29%), pulmonary tuberculosis (3.29%), nontuberculous mycobacteria (1.31%), pulmonary aspergillosis (4.61%) and lung abscess (0.66%). Non-infectious diseases included lung cancer (26.97%), pulmonary nodules (1.97%), sarcoidosis (2.63%), bronchial foreign bodies (0.66%), bronchial stenosis (1.32%), and hemoptysis of unknown cause (0.66%), without concurrent infection. When multiple respiratory diseases coexisted in a patient, the classification was based on the patient’s primary diagnosis and the purpose of bronchoscopy. 70.39% of the patients were given intravenous or oral antibiotic therapy at the same time as bronchoscopy.

### 3.2 Changes of aerosol microbial concentration before and after bronchoscopy

The total concentration of aerosol microorganisms after bronchoscopy was 89.60 ± 63.52 CFU/m^3^, which was significantly higher than 43.80 ± 26.70 CFU/m^3^ before bronchoscopy. The concentrations of bacteria and fungi after bronchoscopy were, respectively, higher than those before bronchoscopy, and the differences were statistically significant (Table 2) (*p* < 0.001) while a part of microorganisms that could not be identified were excluded because colonies were too small, fused, contaminated, etc. The aerosol bacteria and fungi before and after bronchoscopy were all obtained by culture on blood agar plates, chocolate agar plates, MacConkey agar plates, and anaerobic blood agar plates.

**Table 2:** Aerosol microbial concentration before and after bronchoscopy.

Group	*n*	Pre-bronchoscopy (CFU/m^3^)	Post-bronchoscopy (CFU/m^3^)	*p* value
Total	40	43.80 ± 26.70	89.60 ± 63.52	<0.001
Bacteria	40	33.00 ± 20.55	64.05 ± 48.23	<0.001
Fungi	40	9.70 ± 6.89	24.00 ± 15.52	<0.001

The increase in microbial concentration of aerosols was in consistency with the total number of patients undergoing bronchoscopy (*p* = 0.005) and the number of patients with respiratory infectious diseases (*p* < 0.001) on the same day (Table 3).

**Table 3:** The increase in microbial concentration and the number of patients.

Group	*n*	The number of patients	The increase in microbial concentration (CFU/m^3^)	*p* value
Total	40	3.80 ± 1.14	45.80 ± 48.67	0.005
Infectious	40	2.50 ± 1.60	45.80 ± 48.67	<0.001
Non-infectious	40	1.30 ± 1.49	45.80 ± 48.67	0.088

Patients who underwent bronchoscopy on the same day were regarded as a group. Of the 40 patient groups, a total of 18 groups contained only respiratory infectious diseases and 6 groups contained only non-infectious diseases. After bronchoscopy for patients with infectious diseases, the microbial concentration in the air was significantly higher than that for patients with non-infectious diseases (*p* = 0.027) while there was no significant difference between the number of patients in the two groups (*p* = 0.922) (Table 4).

**Table 4:** The microbial concentration for patients with infectious/non-infectious diseases.

Group	*n*	The number of patients	The increase in microbial concentration (CFU/m^3^)
Infectious	18	3.72 ± 1.18	75.44 ± 67.29
Non-infectious	6	3.67 ± 1.21	31.00 ± 24.16
*p* value		0.922	0.027

### 3.3 Species and numbers of aerosol microorganisms before and after bronchoscopy

Before bronchoscopy, the bacteria accounted for 75.34% of the total aerosol microorganisms, fungi 22.17%, and others could not be identified 2.51%. After bronchoscopy, the bacteria accounted for about 70.35% of the total aerosol microorganisms, fungi 26.80%, and other unknown ones 2.85% (Table 5). The bacteria mainly included *Micrococcus luteus*, *Staphylococcus epidermidis*, *Staphylococcus hominis*, *Haemophilus influenzae*, *Neisseria faunalis*, *Staphylococcus capitis*, etc. The fungi mainly included *Aspergillus flavus*, *Aspergillus niger*, *Saccharomyces albicans*, *Penicillium* spp., etc.

**Table 5:** Distribution of the aerosol bacteria and fungi before and after bronchoscopy (culture).

Category	Genus	Species	Before	Percentage (%)	After	Percentage (%)
Gram-positive Cocci	*Micrococcus*	*M. luteus*	145	16.55	247	13.79
	Coagulase-negative *Staphylococci*	*S. epidermidis*	61	6.96	103	5.75
		*S. hominis*	59	6.74	113	6.31
		*S. capitis*	55	6.28	92	5.14
		*S. haemolyticus*	41	4.68	71	3.96
		*S. cohnii*	37	4.22	25	1.40
	Coagulase-positive *Staphylococci*	*S. aureus*	6	0.68	48	2.68
	*Enterococcus*	*Enterococcus faecalis*	1	0.11	1	0.06
	*Streptococcus*	*S. pneumoniae*	8	0.91	91	5.08
Gram-negative Cocci	*Neisseria*	*N. subflava*	56	6.39	77	4.30
		*Neisseria* sp.	4	0.46	0	0.00
Gram-positive Bacilli	*Bacillus*	*Bacillus* spp.	23	2.63	30	1.68
		*Bacillus subtilis*	19	2.17	28	1.56
		*Clostridium* sp.	9	1.03	2	0.11
	*Exiguobacterium*	*Microbacterium* spp.	22	2.51	29	1.62
	*Kocuria*	*Kocuria marina*	21	2.40	24	1.34
		*Kocuria palustris*	15	1.71	6	0.34
	*Arthrobacter*	*Arthrobacter* sp.	12	1.37	1	0.06
Gram-negative Bacilli	*Pseudomonas*	*P. aeruginosa*	9	1.03	44	2.46
	*Pseudoxanthomonas*	*Pseudomonas xanthomarina*	22	2.51	32	1.79
	*Acinetobacter*	*A. baumannii*	10	1.14	32	1.79
	*Klebsiella*	*K. pneumoniae*	0	0.00	7	0.39
	*Escherichia*	*Escherichia coli*	3	0.34	30	1.68
	*Enterobacter*	*Enterobacter cloacae*	8	0.91	23	1.28
		*Enterobacter aerogenes*	3	0.34	14	0.78
	*Haemophilus*	*H. influenzae*	4	0.46	49	2.74
		*H. parainfluenzae*	1	0.11	33	1.84
	*Rothia*	*Rothia aeria*	6	0.68	4	0.22
	*Stenotrophomonas*	*Stenotrophomonas maltophilia*	0	0.00	4	0.22
Fungi	*Aspergillus*	*A. fumigatus*	98	11.19	210	11.73
		*A. flavus*	33	3.77	82	4.58
		*A. niger*	26	2.97	54	3.02
	*Candida*	*S. albicans*	25	2.85	83	4.63
		*Candida tropicalis*	2	0.23	10	0.56
	*Nakaseomyces*	*Nakaseomyces glabratus*	0	0.00	2	0.11
	*Penicillium*	*P.* spp.	10	1.14	39	2.18
Unknown	Unknown	Unknown	22	2.51	51	2.85
Total			876	100	1791	100

These microorganisms were classified according to category, genus and species. It contained a variety of staphylococci. Considering that coagulase-negative *staphylococci* (e.g., *S. epidermidis, S. hominis, S. capitis, Staphylococcus haemolyticus, Staphylococcus cohnii*) are weak in pathogenicity and have limited clinical significance. *Staphylococci* were additionally divided into coagulase-negative *staphylococci* (CoNS) and coagulase-positive *staphylococci* (CoPS) (*Staphylococcus aureus*).

**Figure 1:**
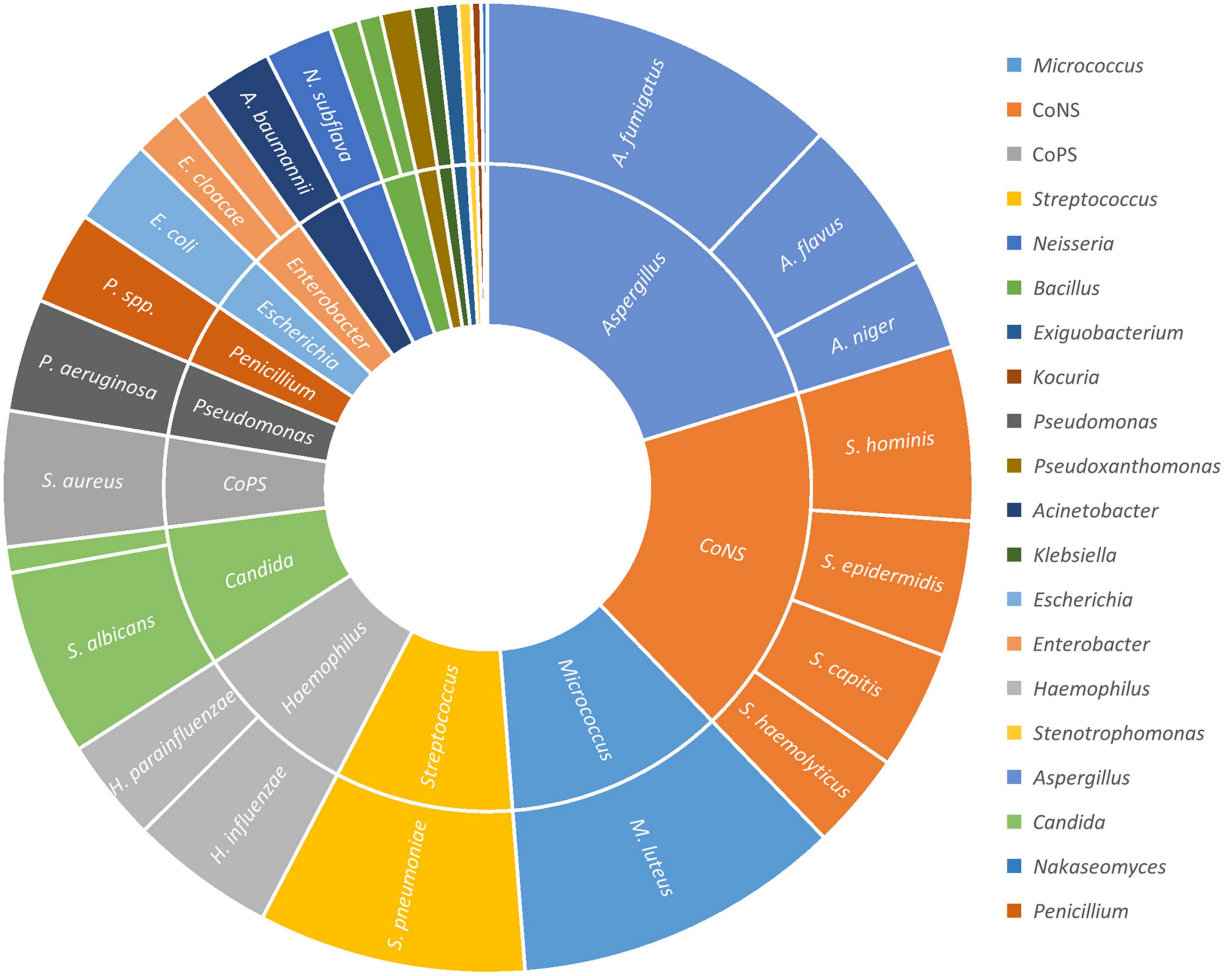
The increase of the aerosol bacteria and fungi after bronchooscopy.

The number of airborne microbial colonies increased from 876 before bronchoscopy to 1791 after bronchoscopy, with a total increase of 915. Among the increased part, *A. fumigatus, S. pneumoniae, S. albicans, A. flavus, H. influenzae,* etc. accounted for a large proportion (Figure 1) (excluding unincreased and unknown species).

### 3.4 Species and numbers of respiratory pathogens in patients with bronchoscopy

Among the 152 patients who underwent bronchoscopy, BALF samples were obtained from 135 of them. Of these, 65 samples were tested by bacterial and fungal culture as well as mNGS, while the remaining 70 samples were only tested by bacterial and fungal culture. And the BALF was not collected in the other 17 patients during bronchoscopy while it was not considered necessary by the operating physician due to the presence of definite diseases such as lung cancer. At least one sputum sample was collected from each one of 152 patients during hospitalization, and a total of 181 sputum samples were collected.

Among the 135 BALF and 181 sputum samples, a total of 148 strains of pathogenic bacteria and fungi [not including *Mycobacterium tuberculosis* (MTB) / non-tuberculous mycobacteria (NTM)] were detected, mainly including *Streptococcus pneumoniae*, *S. albicans*, *H. influenzae*, *Aspergillus fumigatus*, *Haemophilus parainfluenzae*, etc. Most of them were common pathogens of respiratory infection, which was consistent with previous microbial epidemiological statistics (15–17) (Table 6).

**Table 6:** Distribution of the pathogenic bacteria and fungi from patients’ respiratory tracts (culture/mNGS).

Microorganism	Number	Percentage (%)	Only mNGS	Only culture	mNGS +culture
*S. pneumoniae*	18	12.16	4	9	5
*S. albicans*	17	11.49	5	6	6
*H. influenzae*	13	8.78	5	3	5
*A. fumigatus*	12	8.11	6	2	4
*H. parainfluenzae*	12	8.11	4	5	3
*P. aeruginosa*	8	5.41	4	0	4
*S. aureus*	8	5.41	2	3	3
*E. coli*	6	4.05	2	2	2
*A. flavus*	5	3.38	1	2	2
*A. baumannii*	5	3.38	1	1	3
*P.* spp.	5	3.38	0	5	0
*K. pneumoniae*	5	3.38	1	1	3
*C. tropicalis*	4	2.70	1	1	2
*E. aerogenes*	3	2.03	1	1	1
*E. cloacae*	3	2.03	1	1	1
*E. faecalis*	2	1.35	0	0	2
*A. niger*	2	1.35	1	0	1
*Actinomyces odontolyticus*	2	1.35	1	0	1
*S. maltophilia*	2	1.35	1	0	1
*Streptococcus mitis*	2	1.35	1	0	1
*Prevotella melaninogenica*	1	0.68	1	0	0
*Elizabethkingia anophelis*	1	0.68	1	0	0
*Bordetella Pertussis*	1	0.68	1	0	0
*Exophiala oligosperma*	1	0.68	1	0	0
*N. glabratus*	1	0.68	1	0	0
*Serratia marcescens*	1	0.68	1	0	0
*Enterococcus faecium*	1	0.68	0	1	0
*Enterococcus lactis*	1	0.68	0	0	1
*Enterobacter hormaechei*	1	0.68	1	0	0
*Lautropia mirabilis*	1	0.68	1	0	0
*Candida parapsilosis*	1	0.68	0	0	1
*Streptococcus constellatus*	1	0.68	1	0	0
*Cryptococcus gattii*	1	0.68	1	0	0
*Malassezia restricta*	1	0.68	1	0	0
Total	148	100.00	53	43	52

If the same bacteria were detected simultaneously in the sputum and BALF of Patient 001, whether by culture + culture or mNGS + culture, it was regarded as 1 strain. If one strain was detected only by mNGS, it was recorded in the fourth column (Only mNGS) of Table 6. In the same way, if the bacterium was only cultured, it was recorded in the fifth column (Only culture) of Table 6.

In addition, among the 135 BALF samples, 91 samples that needed to be differentiated for MTB / NTM infection were sent for MTB/RIF testing. Ultimately, 5 samples were MTB/RIF positive (among which 3 samples sent for mNGS all indicated *M. tuberculosis* complex), suggesting MTB infection. Additionally, one case of *Mycobacterium avium* complex and one case of *Mycobacterium abscessus* were both detected by mNGS. No positive results were indicated by culture.

### 3.5 Correlation between increased aerosol pathogens and the respiratory pathogens of patients

**Figure 2:**
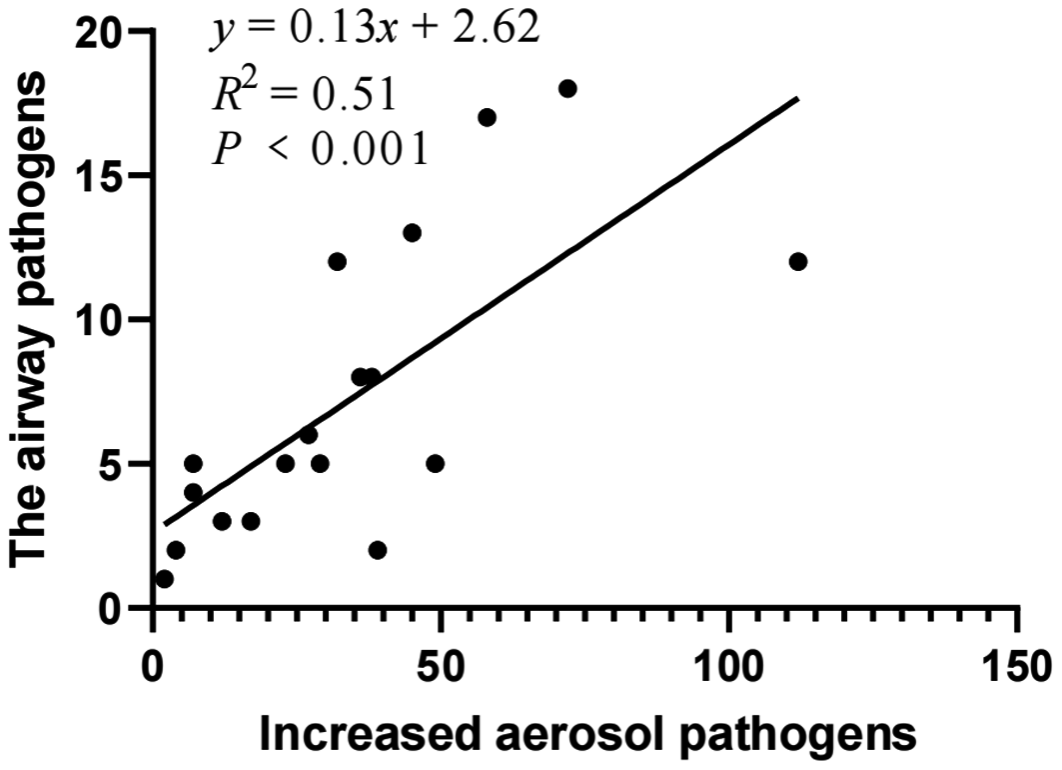
Correlation between increased aerosol pathogens and the respiratory pathogens of patients.

The top 18 pathogens isolated from the respiratory tract of patients were selected and analyzed for the correlation with the increased colony numbers of aerosols (Table 7). *Pearson* correlation analysis was used. The species and numbers of aerosol microorganisms increased after bronchoscopy were moderate correlation with the distribution of pathogenic bacteria and fungi of respiratory infections in patients (*r* = 0.716, *p <* 0.001) (Figure 2).

**Table 7:** Correlation between increased aerosol pathogens and the respiratory pathogens of patients.

Pathogens	Increased colony number (cfu)	Patients’ pathogens (strain)
*A. fumigatus*	112	12
*S. pneumoniae*	72	18
*S. albicans*	58	17
*A. flavus*	49	5
*H. influenzae*	45	13
*A. niger*	39	2
*S. aureus*	38	8
*P. aeruginosa*	36	8
*H. parainfluenzae*	32	12
*P.* spp.	29	5
*E. coli*	27	6
*A. baumannii*	23	5
*E. cloacae*	17	3
*E. aerogenes*	12	3
*C. tropicalis*	7	4
*K. pneumoniae*	7	5
*S. maltophilia*	4	2
*N. glabratus*	2	1
Total	609	129

### 3.6 The rate of nosocomial respiratory infection and the risk assessment

During the study, there were 122 inpatients with bronchoscopy in the general ward of the respiratory department, 3 of whom suffered nosocomial respiratory infections, with a nosocomial infection rate of 2.52%. Meanwhile, there were 572 other inpatients in the general ward of the respiratory department without bronchoscopy, among whom 12 suffered nosocomial respiratory infections, indicating an infection rate of 2.28%. These statistics suggested that the operation of bronchoscopy did not increase the risk of nosocomial respiratory infection of patients (*p* = 0.735) (Table 8).

**Table 8:** The rate and the risk of nosocomial respiratory infection.

	Nosocomial infection	No nosocomial infection	Rate (%)	*p* value
Bronchoscopy	3	119	2.52	0.735
No bronchoscopy	12	560	2.28	

## 4 Discussion

In recent years, the incidence of opportunistic infections in hospitals has gradually increased, especially in patients with immune deficiency, malignant tumors, and organ transplantation, which can lead to invasive aspergillosis, etc., and even death (18–20). Bronchoscopy is widely used in clinical practice. The target patients of bronchoscopy mainly consist of those with various types of pulmonary infections, tumors, bronchiectasis, hemoptysis, unexplained fever, airway foreign bodies, etc. On the one hand, a large proportion of patients have different degrees of pulmonary infection, and carry various pathogens themselves, often complicated with lung structural lesions, various tumors, blood system diseases, long-term bed rest, catheters and other immunosuppressive factors. On the other hand, the bronchoscopy room is a special closed indoor environment with a high degree of spread and contamination of pathogenic microorganisms. During the operation, patients’ respiratory tracts remain open continuously, they cough and even vomit frequently, and their respiratory tract secretions carry a large number of pathogenic microorganisms, which can bring serious pollution to the indoor environment. With a large number of various microorganisms in the air, the environment of the bronchoscopy room may be more complex than other hospital environments.

Since pathogenic microorganisms are transmitted by aerosol, patients accepting bronchoscopy or others are more prone to secondary infection through inhalation of aerosol due to low immunity, damage of airway mucosal protective layer and their own underlying diseases under this circumstance. Airway opening during bronchoscopy also provides an opportunity for pathogenic bacteria and fungi to invade, because the damage to patients’ airway mucosa can lead to weakened or even lost ability to remove the pathogens, increasing the risk of respiratory infection. Patients’ conditions can be aggravated or even life-threatening due to nosocomial infection, which not only increases the hospital stay and cost, but also greatly raises the mortality rate. In addition, it may also cause cross-infection among medical workers (21, 22).

In dynamic environments such as aerosol-generating operations, controlling the concentration of bacteria and fungi in the air is vital to reduce nosocomial infections caused by aerosol transmission. Therefore, it is one of the most critical measures for the prevention and control of nosocomial infection to formulate an effective air management program in the bronchoscopy room and block the transmission route of infection in the air environment of the bronchoscopy room. The environment, staff, facilities, especially air purification equipment of the bronchoscopy room in different hospitals vary significantly, and it still awaits further study on how to formulate effective individualized air purification programs in each bronchoscopy. Although there are some general standards such as GB15982-2012 “Hygienic Standards for Hospital Disinfection” (14), formulating the hygienic standards for the class I-IV environments of hospitals in static state, there is no unified hygienic standard for the dynamic disinfection of indoor air in hospitals, and the follow-up updates and research data are insufficient. At present, the key of most domestic and foreign research is the effect of terminal disinfection and the influencing factors of indoor air under static conditions.

According to the GB15982-2012 “Hygienic Standards for Hospital Disinfection,” the bronchoscopy room is sterilized and managed based on the standard requirements of class III environment. The total number of air bacterial colony should be ≤500 CFU/m^3^ (air sampler method) or ≤ 4.0 (5 min) CFU/plate (plate exposure method) (14). In this study, the concentration of airborne microorganisms in the bronchoscopy room of our hospital was 43.80 ± 26.70 CFU/m^3^ before and 89.60 ± 63.52 CFU/m^3^ after the operation of the bronchoscopy, which met the standard.

This study innovatively examined the variation of the number and species of airborne microbial colonies under aerosol generation operations. The results show that the number of airborne microorganisms, including bacteria and fungi, can be significantly increased by performing aerosol-generating bronchoscopy, which is consistent with the results of other studies (23–25).

Previous studies have shown that the results of mNGS in BALF are consistent with those of classical microbiological methods, and some studies have even shown that mNGS has higher sensitivity and specificity. In addition, it is more convenient to infect pathogens that are difficult to culture, such as anaerobes, nocardia and low load bacteria (26, 27). In this study, the results of mNGS were also consistent with the results of sputum and BALF culture.

There are a large number of colonization bacteria in the nasopharynx and oropharynx of healthy people, so non-infected patients can also produce aerosols containing microorganisms during bronchoscopy. Routine bronchoscopic procedures for infected patients include bronchoalveolar lavage, bronchial brushing, and biopsy. Routine bronchoscopic procedures for non-infected patients include biopsy, bronchial brushing, bronchoalveolar lavage, ultrasound-guided transbronchial node biopsy, removal of foreign body, transbronchial biopsy, placement/removal of airway stent, argon plasma coagulation, cryotherapy, etc. The results of this study showed that the increase in the number of airborne microorganisms was correlated with the total number of bronchoscopy cases, suggesting that microbe-containing aerosols could be produced no matter what kind of bronchoscopic procedures patients accepted. At the same time, the results showed that the raised number of airborne microorganisms was correlated with the number of patients with respiratory infections. After bronchoscopy, the concentration of airborne microorganisms in patients with respiratory infections was significantly higher than that in non-infected patients. These results suggest that compared with non-infected patients, the aerosols produced by patients with respiratory infectious diseases contain more pathogenic bacteria and fungi. Therefore, if the total number of bronchoscopy operations or the number of patients with respiratory infectious diseases further increases, the dynamic concentration of microbial aerosols may exceed the safety threshold value.

The infection process is the interaction between pathogen concentration (infection dose), virulence and the organism. With diverse sources and complex diffusion, microbial aerosols could lead to widespread infections. Bioaerosols with high concentration and small diameter invaded the body through respiration and other ways, which were more likely to cause respiratory and lung diseases and increase the risk of nosocomial infection (28–30). Yousefzadeh A et al. also found that the overall average hospital air pollution to bioaerosols was slightly higher than the standards proposed by international organizations, and microbial aerosol was a risk factor leading to nosocomial infection of patients, related to the type, concentration and exposure time of aerosol particles (31). A total of 148 strains of pathogenic bacteria and fungi were isolated and detected from the patients’ respiratory tracts in this study, mainly including *S. pneumoniae*, *S. albicans*, *H. influenzae*, *A. fumigatus*, *H. parainfluenzae*, etc. Most of them were common pathogens of respiratory infections, which was consistent with previous microbial epidemiological statistics (15–17, 32).

The microorganisms in the air before the operation of the bronchoscopy were mainly common bacteria in natural environments and conditioned pathogens such as *M. luteus*, *S. epidermidis*, *S. hominis*, *Neisseria subflava*, *S. capitis*, and the pathogenic bacteria strongly associated with respiratory tract infections were rare.

The top 18 pathogens isolated from the respiratory tract of patients were selected and analyzed for the correlation with the increased colony numbers of aerosols. The species and numbers of aerosol microorganisms increased after bronchoscopy were moderate correlation with the distribution of pathogenic bacteria and fungi of respiratory infections in patients.

And the increase of pathogens populations was mainly caused by common respiratory pathogens such as *S. pneumoniae*, *S. albicans*, *H. influenzae* and *S. aureus*, indicating that bronchoscopy operation may lead to subsequent exposure of patients to pathogenic aerosol, which may be one of the factors leading to nosocomial infection.

Among the detected microorganisms, we focused more on those pathogens closely associated with respiratory infections, such as *A. fumigatus, S. pneumoniae, Pseudomonas aeruginosa, Acinetobacter baumannii, Klebsiella pneumoniae* subsp. *pneumoniae, S. aureus*.

*A. fumigatus* is a common opportunistic fungal pathogen capable of causing invasive aspergillosis, particularly in immunocompromised patients. It produces minute conidia that can be easily inhaled into the lungs and rapidly proliferate under suitable conditions. This fungus is one of the leading causes of severe pulmonary infections, posing a significant threat to individuals with compromised immune systems (33).

*S. pneumoniae* is one of the most common pathogens responsible for community-acquired pneumonia. It can cause serious diseases such as meningitis and sepsis. Its surface components, including capsular polysaccharides and proteins, aid in evading host immune responses. This bacterium leads to various invasive diseases, including but not limited to pneumonia, otitis media, and sinusitis (34).

*P. aeruginosa* is a multidrug-resistant bacterium with broad metabolic capabilities, allowing it to survive in diverse environments. It secretes multiple toxins and effector molecules that disrupt cell structures and induce inflammatory responses. Widespread in hospital settings, this bacterium frequently causes chronic respiratory infections, especially in cystic fibrosis patients or those with prolonged hospital stays (35).

Known for its high resistance to antibiotics, particularly carbapenems, *A. baumannii* can form biofilms to resist external stresses and spreads rapidly within healthcare facilities. This bacterium is primarily associated with hospital-acquired infections, such as pneumonia, urinary tract infections, and wound infections (36).

*K. pneumoniae* can produce extended-spectrum *β*-lactamases (ESBLs) and other resistance mechanisms, leading to difficult-to-treat infections. Its capsule enhances adhesion and resistance to phagocytosis. This bacterium causes severe infections both in communities and hospitals, notably pneumonia, bacteremia, and urinary tract infections (37).

Variants like methicillin-resistant *S. aureus* (MRSA) exhibit strong invasiveness and produce multiple toxins, causing a range of conditions from skin and soft tissue infections to endocarditis. *S. aureus* is a significant source of both hospital and community infections, drawing considerable public health attention due to its high antibiotic resistance (38).

These microorganisms are characterized by their potent pathogenicity and potential for drug resistance, warranting special attention in clinical settings. These pathogens can lead to severe health issues and often evade effective control by conventional antibiotics.

In this study, a variety of classic nosocomial microorganisms were detected in air samples after bronchoscopy, including *A. baumannii, P. aeruginosa, K. pneumoniae*, and others. Although the detection rate of these pathogens was relatively low, their presence was of concern because they are closely associated with hospital-acquired pneumonia (HAP), catheter-associated infections, and transmission of resistant bacteria (35–38).

However, our analysis of the rate of nosocomial infection did not show a significant difference between the bronchoscopy group and the control group, which may suggest that current bronchoscopy disinfection procedures and environmental controls (e.g., air filtration, hand hygiene) effectively reduce the risk of transmission of these pathogens. Future studies can further monitor the airborne load of these resistant bacteria and its correlation with clinical infections to optimize infection prevention and control strategies.

Infection is the result of a combination of factors. The nosocomial infection rate of patients with bronchoscopy in this study did not increase, and other factors such as exposure time and body immunity should be comprehensively considered. 70.39% of the patients were given intravenous or oral broad-spectrum antibiotic therapy at the same time as bronchoscopy, which may reduce the nosocomial infection probability. Moreover, due to the small sample size, the use of antibiotics cannot be excluded, which may interfere with the analysis results. Thus, the possibility of nosocomial infection in patients and even medical staff caused by aerosols in the bronchoscopy room cannot be completely ruled out, which should be further studied subsequently.

Although some pathogens are vaccine-preventable such as *S. pneumoniae* and *H. influenzae*, their presence in our study can be explained by the following factors:

Vaccination Coverage Gaps: In our country (China), pneumococcal and Hib (*Haemophilus influenzae* type b) vaccines are included in the national immunization program, but coverage is not universal (especially in older adults and immunocompromised patients). Some individuals may remain unvaccinated or have waning immunity.

Non-Vaccine Serotypes/Strains: Current vaccines (e.g., PCV13, Hib conjugate vaccine) do not cover all serotypes. For example, *S. pneumoniae* has over 90 serotypes, and non-vaccine strains can still colonize and transmit.

Asymptomatic Carriage: Even vaccinated individuals can carry these bacteria in the nasopharynx without symptoms, potentially shedding them into the environment during procedures like bronchoscopy.

Immunocompromised Patients: Bronchoscopy is often performed on high-risk patients (e.g., those with COPD, cancer, or immunosuppression), who may have reduced vaccine efficacy or higher susceptibility to infections.

Besides, despite vaccination programs, these pathogens remain clinically relevant due to: Antimicrobial Resistance: Some strains (e.g., penicillin-resistant *S. pneumoniae*, *β*-lactamase-producing *H. influenzae*) complicate treatment. Opportunistic Infections: In hospitalized or immunocompromised patients, even vaccine-covered strains can cause severe pneumonia or invasive disease. Environmental Persistence: These microbes can survive on surfaces or in aerosols, contributing to nosocomial transmission risks.

While vaccines reduce the burden of *S. pneumoniae* and *H. influenzae*, their detection in bronchoscopy settings highlights the need for enhanced infection control measures (e.g., air filtration, PPE) to protect vulnerable patients. Future studies could correlate microbial air contamination with patient colonization status and vaccine histories.

In addition, 5 MTB and 2 NTM patients were diagnosed with mNGS and Xpert MTB/RIF. MTB/NTM require special solid or liquid media such as BACTEC MGIT 960, and some grow slowly, taking 2–8 weeks. Considering that conventional bacterial cultures on common agar plates for days cannot identify mycobacteria, it was not discussed in this study whether mycobacterium aerosols existed in the air.

According to the GB15982-2012 “Hygienic Standards for Hospital Disinfection” of the Ministry of Health of the People’s Republic of China, indoor air is directly collected on agar plates in either air sampler method or plate exposure method.

However, viruses cannot be cultured on agar plates and need to be collected using different air samplers and detected by special methods such as mNGS and PCR (39, 40). We did not have enough funds to perform mNGS or PCR tests on every air sample, and we did not have virus collection tools. Also, there is no standard for indoor air virus concentrations. Therefore, aerosols of viruses were not included in this study, although respiratory viruses were included in the mNGS data. At the same time, there is no standard for indoor air virus concentrations.

Fungi are more appropriately cultured on specialized media such as SDA and PDA (41–43). The low pH and specific nutrients in these media effectively inhibit bacterial growth while promoting optimal fungal development. Meanwhile, optimal growth temperatures for different microorganisms vary (44).

The combination of blood agar plates, chocolate agar plates, MacConkey agar plates and anaerobic blood agar plates is a versatile medium capable of supporting the growth of a wide range of aerosol microorganisms, including the vast majority of bacteria and some fungi. In order to maintain the consistency of experimental conditions, we chose 36 ± 1°C, which is suitable for the growth of most bacteria and some fungi. It is closer to the human environment and helps reflect the microbial communities in indoor air that are relevant to human health. But it may lead to underestimation of the number and species of fungi.

SDA/PDA is considered to be used for future studies such as drug resistance in fungi to further validate and complement our findings.

This study provides a scientific basis for further developing a novel, safe, practical and effective air management method in the bronchoscopy room, offers a reference to the hygienic standard of dynamic disinfection of indoor air in hospitals, and casts new light on preventing nosocomial infection. The era of precise and individualized treatment also requires precise and individualized disinfection management measures.

In summary, bronchoscopy operation can increase the concentration of aerosol microorganisms. The increased microorganisms are correlated with patients’ respiratory pathogens, which are mainly common respiratory pathogens, but the bronchoscopy operation does not increase the risk of nosocomial respiratory infection of patients.

## Data Availability

The original contributions presented in the study are included in the article/Supplementary material, further inquiries can be directed to the corresponding author.
