# Supplementary material for: Distribution characteristics of aerosol microorganisms in bronchoscopy room and the risk assessment of nosocomial infection
Source: Front Public Health. 2025 Apr 28;13:1556364. doi: 10.3389/fpubh.2025.1556364 (PMC12066432; doi:10.3389/fpubh.2025.1556364)
Supplement: Supplementary file 1 [file Table_1.docx]

Supplementary Material

# Supplementary Tables

**Table 1S.** The increase of the aerosol bacteria and fungi after bronchoscopy

| **Microorganism** | **The increase** | **Percentage(%)** |
| --- | --- | --- |
| *Staphylococcus capitis* | 112 | 12.23 |
| *Micrococcus luteus* | 102 | 11.14 |
| *Acinetobacter baumannii* | 72 | 7.86 |
| *Pseudomonas xanthomarina* | 58 | 6.33 |
| *Staphylococcus epidermidis* | 54 | 5.90 |
| *Microbacterium* spp. | 49 | 5.35 |
| *Enterococcus faecalis* | 45 | 4.91 |
| *Neisseria subflava* | 42 | 4.59 |
| *Pseudomonas aeruginosa* | 39 | 4.26 |
| *Staphylococcus hominis* | 38 | 4.15 |
| *Enterobacter cloacae* | 38 | 4.15 |
| *Haemophilus influenzae* | 36 | 3.93 |
| *Aspergillus flavus* | 32 | 3.49 |
| *Bacillus* spp. | 30 | 3.28 |
| *Saccharomyces albicans* | 29 | 3.17 |
| Unknown | 29 | 3.17 |
| *Klebsiella pneumoniae subsp. pneumoniae* | 27 | 2.95 |
| *Rothia aeria* | 23 | 2.51 |
| *Staphylococcus haemolyticus* | 21 | 2.29 |
| *Escherichia coli* | 17 | 1.86 |
| *Aspergillus fumigatus* | 12 | 1.31 |
| *Bacillus subtilis* | 10 | 1.09 |
| *Kocuria marina* | 7 | 0.76 |
| *kocuria palustris* | 7 | 0.76 |
| *Stenotrophomonas maltophilia* | 7 | 0.76 |
| *Penicillium* spp. | 7 | 0.76 |
| *Candida tropicalis* | 4 | 0.44 |
| *Arthrobacter* sp. | 3 | 0.33 |
| *Clostridium* sp. | 2 | 0.22 |
| *Nakaseomyces glabratus* | 2 | 0.22 |
| *Aspergillus niger* | 0 | 0.00 |
| *Enterobacter aerogenes* | -2 | -0.22 |
| *Haemophilus parainfluenzae* | -4 | -0.44 |
| *Streptococcus pneumoniae* | -6 | -0.66 |
| *Neisseria* sp. | -6 | -0.66 |
| *Staphylococcus aureus* | -8 | -0.87 |
| *Staphylococcus cohnii* | -12 | -1.31 |
| Total | 916 | 100.00 |
